# Effects of carbon-to-sulfur (C/S) ratio and nitrate (N) dosage on Denitrifying Sulfur cycle-associated Enhanced Biological Phosphorus Removal (DS-EBPR)

**DOI:** 10.1038/srep23221

**Published:** 2016-03-17

**Authors:** Mei Yu, Hui Lu, Di Wu, Qing Zhao, Fangang Meng, Yudan Wang, Xiaodi Hao, Guang-Hao Chen

**Affiliations:** 1 School of Environmental Science and Engineering, Sun Yat-sen University, Guangzhou, 510275, China; 2 Guangdong Provincial Key Laboratory of Environmental Pollution Control and Remediation Technology (Sun Yat-sen University), Guangzhou, 510275, China; 3 Department of Civil and Environmental Engineering, The Hong Kong University of Science and Technology, Clear Water Bay, Hong Kong, China; 4 School of Civil Engineering, Guangzhou University, Guangzhou, China; 5 Shenzhen Fengrun Environmental Technology Co., Ltd, Shenzhen, China; 6 Key Laboratory of Urban Stormwater System and Water Environment -MoU/R and D Centre for Sustainable Wastewater Treatment, Beijing University of Civil Engineering and Architecture, Beijing 100044, P. R. of China

## Abstract

In this study, the Denitrifying Sulfur cycle-associated Enhanced Biological Phosphorous Removal (DS-EBPR) with 20 mg P/L/d of the volumetric P removal rate was successfully achieved in a Sequencing Batch Reactor (SBR). The effects of carbon-to-sulfur (C/S) mass ratio and nitrate (N) dosage were investigated through two batch tests to reveal the role of wastewater compositions in DS-EBPR performance. The optimal specific P release and uptake rates (0.4 and 2.4 mg P/g VSS/h, respectively) were achieved at C/S/P/N mass ratio of 150/200/20/20, and poly-S is supplied as a potential electron and energy storage. The nitrate dosage in a range of 10–50 mg N/L had no significant influence on P uptake rates (2.1 ~ 2.4 mg P/g VSS/h), but significantly affected the storage of inclusion poly-S, the poly-S oxidation rate was increased about 16% while dosing nitrate from 20 to 30 mg N/L. It implies that nitrate is denitrified in the P uptake phase, and excess nitrate is further consumed by poly-S. Moreover, the microbial analysis showed that the functional bacteria should mostly belong to denitrifying bacteria or *Unclassified* genera.

A novel Sulfate reduction, Autotrophic denitrification and Nitrification Integrated (SANI) process has been developed to treat Hong Kong’s saline sewage resulting from half century practice of seawater toilet flushing^1^. In the process, sulfate, one of the major constituents of seawater, has connected efficient carbon conversion via sulfate reducing bacteria (SRB) with autotrophic denitrification via sulfide oxidation bacteria (SOB) for simultaneous organic and nitrogen removal^2^. And a pilot study, which compared with conventional biological nutrients removal (BNR) processes, confirmed 90% reduction of biological sludge production, 35% energy saving and 36% reduction of CO_2_ emission compared with conventional biological nutrients removal (BNR) processes^3^,^4^. Moreover, the SANI process has recently been successfully scaled up to 1,000 m^3^/d for municipal wastewater treatment at the Sha Tin Sewage Treatment Works in Hong Kong, China.

Denitrifying Sulfur cycle-associated Enhanced Biological Phosphorus Removal (DS-EPBR) process was developed to apply the SANI process in phosphorus-limiting estuary regions for controlling eutrophication^5^,^6^. In the early development of DS-EBPR process, nitrate dosage was applied to replace limited oxygenation for integrating denitrification and P removal, which efficiently reduced the cycle time of Sequencing Batch Reactor (SBR) from 42 h to 24 h at 20 °C^6^. However, there are still some problems limiting the application of DS-EBPR process. For instance, the cycle time of DS-EBPR process is still too long as compared with that of the conventional denitrifying EBPR process which could finish one cycle in less than 6 h^7^. More importantly, the role of nitrate in P uptake and release of the DS-EBPR process was not well revealed. It should be noted that the nitrate concentration is the key factor affecting the DS-EBPR process, e.g., a high nitrate concentration could decrease the P release and make the reactor eventually collapse during a long-term operation^8^. Additionally, there is little investigation to reveal what kind of microbial mechanism and metabolism involved in the DS-EBPR process, particularly for the bacteria responsible for denitrification in anoxic P uptake^6^.

The problems mentioned above are closely related with the electron transfer and storage during the biological P removal. For instance, in the conventional EBPR process, the volatile fatty acids (VFA) and nitrate, as the electron donor and acceptor respectively, could directly affect the poly-β-hydroxyalkanoate (PHA) storage and utilization, the P release and uptake rates, the secondary P release and the hydraulic retention time (HRT) via the electron transfer and storage^9^,^10^. Therefore, the acetate (electron donor), nitrate (electron acceptor) and intracellular poly-S (possibly associating the electron transfer and energy storage) in the DS-EBPR process are certainly the essential subjects in the DS-EBPR study.

Although this study does not target to figure out the microbial mechanism of DS-EBPR thoroughly, it is proposed to: (1) optimize the DE-EBPR process and improve the overall performance including the cycle time of SBR, the P removal rate and the secondary P release etc.; (2) reveal the role of influent C/S mass ratio and nitrate dosage on the DS-EBPR and provide insight into the sulfur association function via poly-S storage and oxidation in the DS-EBPR through the batch tests; and (3) examine the diversity and composition of microbial community in the DS-EBPR reactor with both 454-pyrosequencing analysis and fluorescence *in situ* hybridization (FISH) for cross-checking.

## Results

### SBR operation and overall performance

A tightly sealed SBR with 10 L working volume was set-up and continuously operated for over 310 days at the controlled temperature of 22 ± 2 °C in the laboratory (Fig. S1). The concentrations of acetate, sulfate and phosphate in raw synthetic wastewater were 150 ± 7 mg C/L, 200 ± 8 mg S/L and 20 ± 1 mg P/L, respectively, nitrate was lower than 2.0 mg N/L, and pH was 7.1 ± 0.1. The whole experimental period can be divided into three stages as shown in Table 1: Stage I (Day 1 ~ 90): system set-up and inoculation with SRT = 100 days and P release/uptake time = 11/12 h; Stage II (Day 91 ~ 180): SBR operation optimization with SRT = 90 days and P release/uptake time = 9/4 h; and Stage III (Day 181 ~ 310): steady-state operation with SRT = 90 days and P release/uptake time = 6/2 h.

The overall performance of SBR during the whole experimental period was summarized in Table 2, and the P release, P uptake and P removal performance were shown in Fig. S1 of Supplementary Information (SI). Obviously, the volumetric P removal rate in the SBR was significantly improved by about 9 times from 2.3 mg P/L/d in Stage I to 20 mg P/L/d in Stage III and the secondary P release was below 1mg P/L, which was defined as the P release occurring in the absence of VFA while nitrate has been complete denitrified^11^. Moreover, the microbial biomass concentration and sludge P content in the SBR were also increased from 3.3 g/L VSS with 16 ~ 28 mg P/g VSS P content to 3.6 g/L VSS with 40 ~ 70 mg P/g VSS P content as shown in Table 2.

The performance of DS-EBPR process, whose volumetric acetate uptake rate (175 mg C/L/d), sulfate reduction rate (85 mg S/L/d) and P removal rate (20 mg P/L/d) were about 3 times of those in a previous study (58 mg C/L/d, 27 mg S/L/d and 7 mg P/L/d, respectively), and the cycle time (9 h/cycle in Stage III) was only ~1/3 of that in a previous DS-EBPR (24 h)^6^. However, both volumetric P removal rate and cycle time were quite closed to the values (30 mg P/L/d and 6 h/cycle) in a conventional denitrifying EBPR^7^, although the volumetric acetate uptake rate and nitrate denitrification rate were still much lower than those in the conventional denitrifying EBPR as listed in Table 3.

The P content in biomass of 40 ~ 70 mg P/g VSS was achieved in this study at 22 °C and a previous study at 20 °C by Wu *et al*.^6^, even it was still lower than that of conventional denitrifying EBPR at 22 °C (90 ~ 170 mg P/g PAO-VSS)^7^. The sludge yield in this study was only 0.10 ± 0.02 g VSS/g COD that was much lower than that of the conventional denitrifying EBPR process (0.18 ~ 0.21 g VSS/g COD), and close to the biological sludge yield coefficient in the sulfur-cycle involved SANI process (0.02 g VSS/g COD reported by Lu *et al*.^4^), indicating that the DS-EBPR process was effective on minimizing biological sludge production. The secondary P release could be caused by the intracellular poly-P degradation to supply energy (ATP) for microbial maintenance^7^,^12^, which could deteriorate the P removal efficiency and cause the reactor eventually to collapse during long term operation^8^. The secondary P release at Stage III of this study (<1 mg P/L) was obviously lower than that in previous study (4 ± 2.2 mg P/L)^6^, which could be one reason for the higher volumetric P removal rate achieved in this study compared to the previous process. Also the lower secondary P release in this study may be caused by the different influent C/S mass ratios and nitrate dosages relative to Wu *et al*.^6^ as shown in Table 3. So the effects of influent C/S mass ratio and nitrate dosage were further investigated through batch tests as discussion below.

### Effect of Influent C/S mass ratio on DS-EBPR and inclusion poly-S

**Figure 1:**
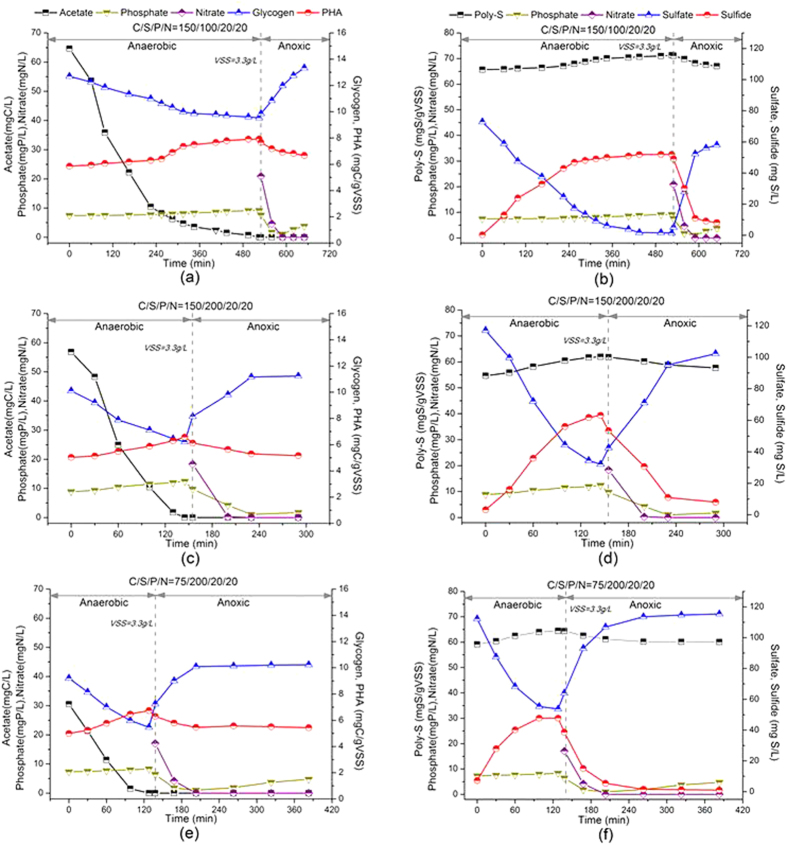
Influent C/S mass ratio effect on DS-EBPR in the Batch Test I: (**a**,**c**,**e**) on the left side: concentration profiles of acetate, phosphate, nitrate, glycogen and PHA; (**b,d**,**f**) on the right side: concentration profiles of sulfate, sulfide, phosphate, nitrate, and poly-S.

The Batch Test I was designed to investigate the effect of influent C/S mass ratio on DS-EBPR with the same P content and nitrate dosage (20 mg P/L and 20 mg N/L) and acetate as the carbon source. Based on the concentration of influent acetate and sulfate of the sulfate reduction bioreactor of the SANI pilot plant (~150 mg C/L, ~200 mg S/L) corresponding to C/S mass ratio of 150/200 mg C/mg S^4^, and the influent C/S mass ratio of 150/100 mg C/mg S was based on that non-sulfate reducing pathway was resulted when influent C/S mass ratio exceeded 1.4, also the influent C/S mass ratio of 75/200 mg C/mg S was based on that sulfate reducing pathway was resulted while the ratio was less than 0.6, and there existed competition of two pathways (i.e. non-sulfate and sulfate reducing pathways) between the ratios of 0.6 and 1.4^13^. The influent acetate and sulfate concentrations of 75–150 mg C/L and 100–200 mg S/L, corresponding to respective C/S mass ratios of 150/100, 150/200 and 75/200, were adopted in Batch Test I. The results of Batch Test I were summarized in Fig. 1 including variation of carbon, sulfur, phosphate and nitrate with organic inclusions (three figures on left side) and poly-S inclusion (three figures on right side) at different C/S/P/N mass ratios. It was shown that the different influent C/S mass ratios resulted the obviously different anaerobic and anoxic time, which was determined by the exhausting time of acetate in anaerobic P release phase and the denitrification time of nitrate in anoxic P uptake phase. And pH of the cycle ranged from 7.3 to 8.6 during the Batch Test I, with the initial pH 7.6. The specific rates of P release/uptake, PHA storage/utilization and poly-S storage/oxidation were determined by the linear regression of linear part of concentration profile with time. As calculated from Fig. 1, the highest anaerobic P release (0.45 mg P/g VSS/h) and the anoxic P uptake (2.4 mg P/g VSS/h) rates were achieved at the C/S mass ratio of 150/200 mg C/mg S. Moreover, interestingly, at the C/S mass ratio of 150/200 mg C/mg S, the lowest PHA storage rate (0.64 mg C/g VSS/h) and the highest P release rate (0.45 mg P/g VSS/h) were found compared with those at C/S mass ratios of 150/100 and 75/200 mg C/mg S in the anaerobic P release phase. Furthermore, in the anoxic P uptake phase, the lowest PHA utilization rate (0.67 mg C/g VSS/h) and the highest P uptake rate (2.4 mg P/g VSS/h) were similarly achieved at the C/S mass ratio of 150/200 mg C/mg S.

The different sulfur compounds including sulfate, sulfite, thiosulfate, sulfide and poly-S were regularly measured during the batch test and shown in Fig. 1 (three figures on right side), in which sulfite and thiosulfate concentrations were quite low and neglected. It was found that the highest poly-S storage and oxidation rates were achieved at the C/S mass ratio of 150/200 mg C/mg S, i.e. 3.0 mg S/g VSS/h and 2.4 mg S/g VSS/h, respectively, compared with C/S mass ratios of 150/100 and 75/200 mg C/mg S. Accordingly, the highest sulfate reduction and generation rates were also found as 10.7 mg S/g VSS/h and 13.5 mg S/g VSS/h at the C/S mass ratio of 150/200 mg C/mg S. The secondary P release was also observed in this batch test and its rate varied significantly with different C/S mass ratios, i.e. it was only 0.2 mg P/g VSS/h at the C/S mass ratio of 150/200 mg C/mg S, lowest compared with C/S mass ratios of 150/100 and 75/200 mg C/mg S (Fig. 1).

There were the lowest PHA utilization rate and highest P uptake rate with the C/S mass ratio of 150/200 mg C/mg S, which was quite unusual to the conventional EBPR as conventional PAO utilized the energy generated from the anaerobic P release phase for PHA storage as well as the energy from PHA utilization for the anoxic P uptake phase, i.e. the highest P release/uptake rate should accompany with the highest PHA storage/utilization rate^10^. So, there should be another inclusion as energy source besides PHA for P release and uptake in the DS-EBPR. What’s more, there were the highest inclusion poly-S storage and oxidation rates with the C/S mass ratio of 150/200 mg C/mg S, and some studies also reported that intracellular sulfur globules could act as temporary energy reservoir and a buffer of electron donating potential in some phototrophic sulfur bacteria^14^.

The energy balance was analyzed based on the metabolic model developed by Smolders *et al*.^15^,^16^ and the Gibbs free energy of chemical reactions as shown in Table 4 and SI. In P release phase of DS-EBPR, 0.04 mmol ATP/g VSS and 0.17 mmol ATP/g VSS were generated in degradation of poly-P and glycogen, respectively. However, only 0.13 mmol ATP/g VSS is stored in PHA production, which implied the energy surplus was 0.08 mmol ATP/g VSS and possibly stored in the poly-S generation (0.09 mmol ATP/g VSS^17^,^18^). In P uptake phase, 0.12 mmol ATP/g VSS and 0.36 mmol ATP/g VSS were stored in generation of poly-P and glycogen, respectively. And PHA degradation could only supply about 0.04 mmol ATP/g VSS. Therefore, the energy deficit was about 0.44 mmol ATP/g VSS that is possibly supplied by the poly-S degradation (0.66 mmol ATP/g VSS^19^).

### Effect of nitrate dosage on DS-EBPR and inclusion poly-S

**Figure 2:**
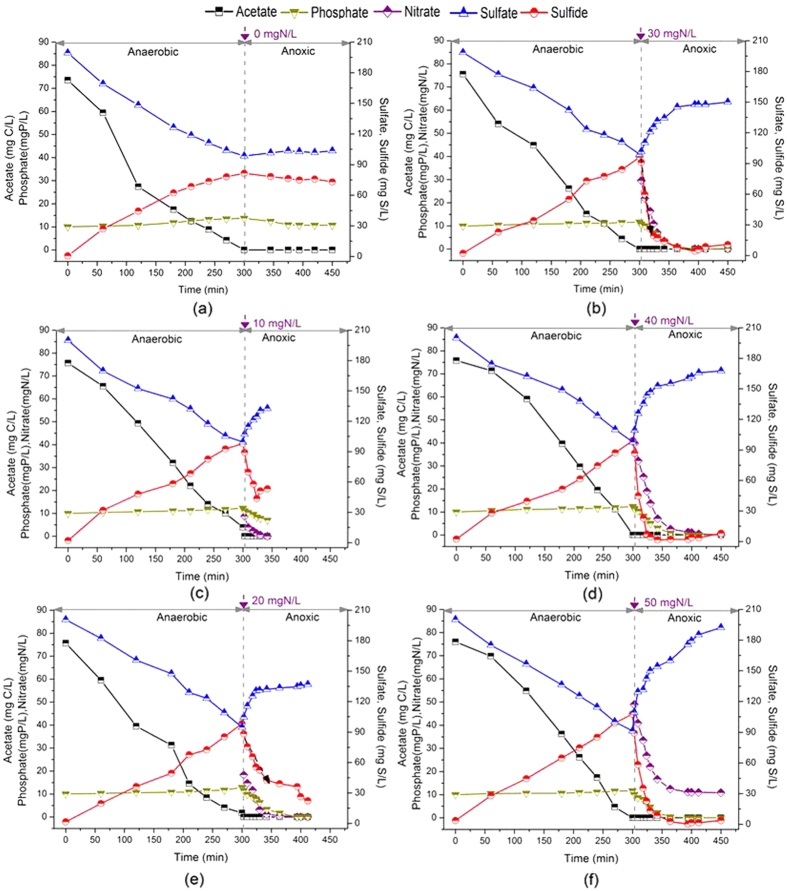
Effect of nitrate dosage at beginning of anoxic phase (purple arrow at the top of each figure as shown) on the DS-EBPR at the same influent C/S/P mass ratio of 150/200/20 mg C/mg S/ mg P in the Batch Test II: (**a**) nitrate dosage = 0 mg N/L; (**b**) nitrate dosage = 10 mg N/L; (**c**) nitrate dosage = 20 mg N/L; (**d**) nitrate dosage = 30 mg N/L; (**e**) nitrate dosage = 40 mg N/L; and (**f**) nitrate dosage = 50 mg N/L.

The Batch Test II was developed to investigate the effect of different nitrate dosage on the DS-EBPR process. Totally five parallel batch experiments were conducted simultaneously with the same C/S/P mass ratio of 150/200/20 mg C/mg S/mg P, but different nitrate dosages (0, 10, 20, 30, 40 and 50 mg N/L) at the beginning of anoxic P uptake phase. And pH of the cycle ranged from 7.2 to 8.8 during the Batch Test II, with the initial pH 7.6. As the anaerobic P release phase were almost the same for five parallel batch tests, it was achieved the similar acetate consumption rate (4.3 mg C/g VSS/h), P release rate (0.15 mg P/g VSS/h), and sulfate reduction rate (5.9 mg S/g VSS/h) as shown in Fig. 2. It is also noted that the similar P uptake rates (2.1 ~ 2.4 mg P/g VSS/h) were achieved even with different nitrate dosages from 10 mg N/L to 50 mg N/L for the anoxic P uptake. However, the phosphate residual (~7 mg P/L) was found with the nitrate dosage 10 mg N/L and the nitrate residual (~11 mg N/L) with the nitrate dosage 50 mg N/L. What’s more, there was more than 10 mg P/L phosphorus residual in the controlled test (0 mg N/L nitrate dosage).

**Figure 3:**
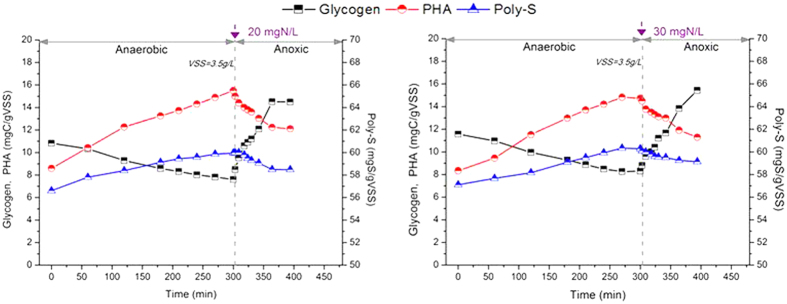
The profiles of intracellular glycogen, PHA and poly-S at the different nitrate dosages in Batch Test II: 20 mg N/L (left) and 30 mg N/L (right).

The variation of biomass inclusion including glycogen, PHA and poly-S was also analysed for the nitrate dosages of 20 mg N/L and 30 mg N/L (Fig. 3). As shown in Fig. 3, the similar PHA utilization (~2.2 mg C/g VSS/h) and glycogen storage (~4.5 mg C/g VSS/h) rates were found for the nitrate dosages of 20 mg N/L and 30 mg N/L in the anoxic P uptake phase. But as showed in Fig. 2, sulfide concentration ranged from 82 mg S/L to 108 mg S/L at the end of anaerobic phase. The dissolved sulfide and poly-S oxidation rates were calculated based on the linear regression of linear part of the concentration profile in Figs 2 and 3 at nitrate dosage of 20 mg N/L and 30 mg N/L^20^. The higher nitrate dosage increased the sulfide and poly-S oxidation rates, i.e. the sulfide and poly-S oxidation rates were 11.3 mg S/g VSS/h and 0.95 mg S/g VSS/h respectively at 20 mg N/L of nitrate dosage, but 16.7 mg S/g VSS/h and 1.1 mg S/g VSS/h respectively at 30 mg N/L.

The P and S mass balance of Batch Test I and II were also analyzed. The average P recovery efficiency was 91.9 ± 1.8%, which was calculated based on the initial and effluent phosphate concentrations, P content in activated sludge, P precipitation and the P loss caused by sampling during batch tests. Moreover, it was conformed that there was no chemical P precipitation and all the P removal was caused by the biological denitrifying P removal based on the P mass balance and content analysis as reported in our previous study^5^. The average S recovery efficiency was 89.3 ± 5.9%, which was calculated by the initial and final concentrations of sulfate, sulfite, thiosulfate, sulfide and poly-S, as well as the S loss caused by sampling during the batch tests.

### Microbial community

The diversity of microbial community in sludge sample taken from the SBR on Day 200 was investigated by 454-pyrosequencing analysis with 8598 sequences acquired after quality filtering in this study and the relative community abundances of the bacterial class level were shown in the pie chart Fig. S4 of SI. Generally, *Candidatus* Accumulibacteria phosphatis (*Accumulibacteria*)^21^ and *Tetrasphaera*^22^ were considered as the major PAO genera in the conventional EBPR system^23^. However, both *Accumulibacteria* and *Tetrasphaera* were detected with non-existence or extremely low abundance (0.02% for *Tetrasphaera*), and the major glycogen accumulating organisms (GAO) genera, such as *Candidatus* Competibacter phosphatis (*Competibacter*)^24^, was also not found. But the major SRB genera in *Deltaproteobacteria* class, e.g. *Desulfobacterium, Desulfobulbus*, *Desulfobacter*^25^, only accounted for 0.3%, 0.6% and 0.02% respectively. The detected SRB genera were also of lower abundance (*Desulfobulus*-like species (<0.01%) and *Desulfomicrobium*-like species (<0.01%)) than the previous study (*Desulfobulus*-like species (10.6%) and *Desulfomicrobium*-like species (10.5%))^6^. Instead, the denitrifying bacteria genera, such as *Thiobacillus* genera (4.7%)^26^,^27^, *Thioclava* genera (1.1%)^28^, *Paracoccus* genera (3.2%) and *Thauera* genera (24.3%)^29^, and *Unclassified* genera (26.1%) were identified to be the predominant groups in the sludge sample which was not considered and investigated in previous studies.

**Figure 4:**
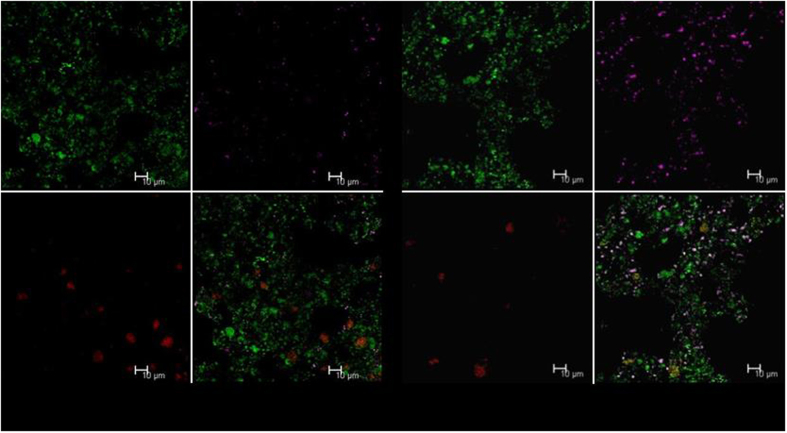
The FISH analysis for composition of microbial community in the SBR: (**a**): Green: all bacteria (EUB), Magenta: conventional poly-phosphate accumulating organisms (PAO) and Red: sulfate reducing bacteria (SRB); and (**b**): Green: EUB, Magenta: denitrifying bacteria and Red: SRB.

The FISH analysis was also conducted to examine diversity and composition of microbial community. Two identical sludge samples were triple-hybridized with different fluorescence probes respectively: Sludge Sample I with FITC (green)-labeled EUBMIX probe, CY5 (red)-labelled SRBMIX probe and CY3 (magenta)-labelled PAOMIX probe, Fig. 4a showed the CLSM (Confocal Laser Scanning Microscope) result that there was abundant total bacteria (green) present, but a little SRB (red) and little PAO (magenta) present; Sludge Sample II with FITC (green)-labeled EUBMIX probe, CY5 (red)-labelled SRBMIX probe and CY3 (magenta)-labelled DMIX probe, Fig. 4b showed the CLSM result that there was abundant total bacteria (green) and denitrifying bacteria (magenta) present, but a little SRB (red) present.

## Discussion

The performance of DS-EBPR process, acetate uptake, sulfate reduction, P removal and cycle time, was better than that of previous study^6^, and P removal and cycle time were quite closed to conventional denitrifying EBPR^7^. The C/S mass ratio and nitrate dosage were found to be relative to higher P removal and lower secondary P release^6^. And the batch tests investigated the effects of influent C/S mass ratio and nitrate dosage. The highest P uptake rate, lowest PHA utilization rate and highest poly-S storage and oxidation rates were got with C/S mass ratio of 150/200 mg C/mg S. The results of energy balance analysis based on the metabolic model during Batch Test I implied that (1) the energy was rich when only considering the PHA as an energy storage for P release and glycogen degradation in anaerobic P release phase; and (2) with only the catabolic energy yield from PHA, the energy was deficient for synthesis of poly-P, glycogen as well as bacteria growth and maintenance. So, it is reasonble and supported by the energy balance analysis that the poly-S (inclusion sulfur) is possibly the potential electron and energy storage as the suppliment of PHA during DS-EBPR process. Generally, the secondary P release process was driven by the energy demand for microorganism maintenance while the external electron acceptor (nitrate) or external carbon source exhausted^7^,^12^,^15^. Thus, the possible reason for the lowest secondary P release rate at C/S mass ratio of 150/200 mg C/mg S in Bath Test I could be the poly-S, since it can act as the potential energy storage as mentioned above and supply metabolic energy for microorganism maintenance during the DS-EBPR process, which was also supported by the highest poly-S oxidation rate (2.4 mg S/g VSS/h) at the C/S mass ratio of 150/200 mg C/mg S.

According to the Batch Test II, it was found that similar specific P uptake rates of 2.1 ~ 2.4 mg P/g VSS/h with the nitrate dosage from 10 mg N/L to 50 mg N/L with the same C/S/P mass ratio of 150/200/20 mg C/mg S/mg P in anoxic P uptake phase. And the inclusion ploy-S stored in the microorganisms was significantly affected by the nitrate dosage during the anoxic P uptake, such as the poly-S oxidation rate increased about 16% while dosing more nitrate from 20 mg N/L to 30 mg N/L. It implies that the nitrate utilization includes two steps during the DS-EBPR process: (1) Nitrate is denitrified in the denitrifying sulfur cycle-associated P removal that has been verified in previous study; and (2) Excess nitrate is further denitrified by the activated microorganisms with inclusion poly-S as the electron donor. Based on the results of Batch Test II as shown in Figs 2 and 3, the optimal nitrate dosage was 20 mg N/L and 30 mg N/L, in which complete P and nitrate removals were simultaneously obtained with the C/S/P mass ratio of 150/200/20 mg C/mg S/mg P.

PHA as the electron and energy storage was not enough to supply the DS-EBPR process from the results of Batch Test I, and poly-S was the potential electron and energy storage confirmed by the energy balance analysis, which showed the mechanisms of DS-EBPR process was different from the conventional EBPR process. During the anoxic phase, nitrate dosage was important to the P removal, and there was influence to the anaerobic phase of P release with excess nitrate dosage, also there would be the secondary P release if nitrate dosage was shortage. From the results of Batch Test II, poly-S could regulate denitrification in the anoxic phase for consuming the excess nitrate and decreasing the secondary P release.

The genera associated with denitrification were identified to be the predominant groups in the sludge sample of this study as mentioned in Results section, the bacteria in the sludge integrating phosphate accumulation and denitrification with sulfur cycle-association could not belong to the conventional PAO, GAO or SRB, but possibly belonged to denitrifying bacteria or *Unclassified* genera. The FISH analysis cross-checked the result of 454-pyrosequencing analysis, and showed that the bacteria performing sulfur cycle-associated P removal in this study could possibly belong to denitrifying bacteria community rather than the conventional PAO or SRB, which is completely different from the conventional EBPR process.

The batch tests and energy balance analysis based on metabolic model confirmed that both poly-S and PHA were the electron and energy storage during DS-EBPR process, and the optimal C/S mass ratio and nitrate dosage were 150/200 mg S/mg C and 20 ~ 30 mg N/L respectively to achieve the highest volumetric P removal rate of 2.4 mg P/g VSS/h and 2.1 ~ 2.4 mg P/g VSS/h. The microbial community in the DS-EBPR reactor was enriched with denitrifying bacteria or *Unclassified* genera and absolutely different from that of the conventional EBPR process enriched with PAO and/or GAO.

## Methods

### SBR set-up and operation

A tightly sealed SBR with 10 L working volume (Fig. S1a) was developed to achieve the DS-EBPR process, which seeded with anaerobic sludge from a saline sewage treatment works in Hong Kong, and continuously operated for about 310 days at controlled temperature of 22 ± 2 °C. The cyclic operation of SBR included four phases as shown in Fig. S1b, e.g. Phase I: 15 min feeding with 5 L synthetic wastewater (composition as shown in Table S1 of SI); Phase II: 6 ~ 11 hours anaerobic P release; Phase III: 2 ~ 12 hours anoxic P uptake with nitrate dosage at the beginning of the phase; Phase IV: 30 min settling; and Phase V: 15 min decanting of 5 L supernatant. There was no purposed sludge withdrawal during whole SBR operation but the regular P harvest by discharging the supernatant at the end of Phase II (P release) for bypass chemical precipitation.

### Batch tests

The activated sludge was taken from the SBR on Day 200 (in Stage III as shown in Table 1) for two types of batch tests investigating the effect of influent C/S mass ratio (Batch Test I) and nitrate dosage (Batch Test II) on the DS-EBPR and the poly-S storage and oxidation during the DS-EBPR. Both types of batch tests were conducted in a set of sealed batch reactors equipped with a magnetic stirrer (200 rpm, IKA) and temperature controlled at 22 ± 2 °C. All batch tests were carried out for three times to check the reproducibility. During the batch tests, 5 mL mixed sample was regularly taken from the batch reactor by a 20 mL syringe equipped with a disposable filter unit (0.45 μm). And then the filtrate was used to determine soluble acetate, phosphate, nitrate, nitrite, sulfate, sulfite, thiosulfate and dissolved sulfide. The unfiltered sludge/solids were immediately inactivated for analyzing PHA, glycogen and poly-S. After each batch test, the remaining sludge by settling in batch reactor was thrown back to the mother SBR.

#### Batch Test I: Effect of influent C/S mass ratio on DS-EBPR and inclusion poly-S

Three batch reactors (1 L and sealed as mentioned above) were parallel conducted to investigate the effect of different influent C/S mass ratios on DS-EBPR and poly-S storage and oxidation as shown in Fig. S1 of SI. Totally 3 L mixed activated sludge was taken from the SBR at the end of P uptake phase and treated by the following steps for three times: (1) washing with distilled water, and (2) settling, to remove the soluble constituents and other possible influences from the mother SBR operation. And then the identical 0.5 L concentrated activated sludge after washing and settling was spiked into each 1 L batch reactor and induced an initial mixed liquor volatile suspended solids (MLVSS) concentration of 6.6 g/L. Subsequently, 0.5 L synthetic influents with different C/S/P mass ratios (150/100/20, 150/200/20, 75/200/20 mg C/mg S/mg P/mg N) were spiked into three batch reactors, respectively. Finally, 20 mg N/L nitrate was dosed into each batch reactor at the end of P release phase for simultaneous anoxic P uptake and denitrification.

### Batch Test II: Effect of nitrate dosage on DS-EBPR and inclusion poly-S

The same batch reactor was used in Batch Tests I and II. In Batch Test II, five identical batch reactors were parallel operated to investigate the effect of nitrate dosage on DS-EBPR process and poly-S storage and oxidation as shown in Fig. S2 of SI. Totally 5 L mixed activated sludge was taken from the SBR at the end of P uptake phase. After the same washing and settling steps as mentioned in Batch Test I, the identical 0.5 L concentrated activated sludge was spiked into each batch reactor and induced an initial MLVSS concentration of 7.0 g/L. And then 0.5 L synthetic influents with the same C/S/P ratio (150/200/20 mg C/mg S/mg P) were spiked into five batch reactors, respectively. After all batch reactors were subjected to P release and P uptake phase with mixing, the different nitrate solutions (0, 10, 20, 30, 40, 50 mg N/L) were dosed into the batch reactors at the beginning of P uptake phase, respectively.

### Analytical methods

The mixed samples regularly grasped from SBR and batch reactors were immediately filtered through a 0.45 μm filter (Jin Teng, China) for the anion concentration analysis, such as soluble acetate, phosphate, sulfate, sulfite, thiosulfate, nitrate and nitrite using ion chromatography (Dionex ICS-900 with IC-AS23 analytical column). Mixed liquor suspended solids (MLSS), MLVSS, and dissolved hydrogen sulfide (H_2_S/HS^−^/S^2−^) were determined according to the Standard Methods^30^. pH and temperature were monitored with a portable pH meter (Mettler Toledo FG2-FK). PHA was determined with high performance liquid chromatography (HPLC, Dionex Ultimate 3000) following the method proposed by De Gelder *et al*.^31^ Glycogen was measured using the anthrone method according to Jenkins *et al*.^32^ And the measurement of poly-S employs the conversion of poly-sulfur and poly-sulfide to thiosulfate at a high pH^33^. Details of the analytical methods of PHA, glycogen and poly-S are shown in SI.

### 454-pyrosequencing analysis and fluorescence *in situ* hybridization (FISH)

The sludge sample taken from the SBR on Day 200 was analyzed by 454-pyrosequencing of 16S rRNA gene (more details in SI), following the protocols reported by Quince *et al*.^34^ and Hao *et al*.^33^, as well as performed for FISH inspection^35^. The sludge sample for FISH analysis was previously fixed overnight in 4% (wt/vol) paraformaldehyde solution at 4 °C, 10~20 μL of each sample was then spotted onto each well of a slide. Prior to the hybridization, samples were dehydrated by sequential immersion in 50, 80 and 100% ethanol for 3 min and air-dried. The 16S rRNA-targeted oligonucleotide probes (Life technologies, Shanghai) (Table S2 of SI) applied on the sludge samples were EUBMIX (EUB338I-III), PAOMIX (PAO462, PAO651 and PAO846), SRBMIX (SRB385d and SRB385Dd), and DMIX (TBD1419, TBD121 and TMD131), and the DMIX probe has all necessary genes that encode the essential enzymes to catalyse the heterotrophic and chemlithotrophic denitrification^36^. The FISH images were obtained from a confocal laser scanning microscope (CLSM) (Leica tcs sp5, Germany) under argon laser (488 nm), DPSS-laser (541 nm) and HeNe-laser (633 nm).

## Additional Information

**How to cite this article**: Yu, M. *et al*. Effects of carbon-to-sulfur (C/S) ratio and nitrate (N) dosage on Denitrifying Sulfur cycle-associated Enhanced Biological Phosphorus Removal (DS-EBPR). *Sci. Rep*. **6**, 23221; doi: 10.1038/srep23221 (2016).

## Supplementary Material

Supplementary Information

## Figures and Tables

**Table 1:** Experimental conditions of the SBR operation (average value  ±  standard deviation).

Stages	Day	SRT^a^ (day)	P release time (h/cycle)	P uptake time (h/cycle)	Initial concentration			
					Acetate^b^ (mg C/L)	Sulfate^b^ (mg S/L)	Phosphate^b^ (mg P/L)	Nitrate^c^ (mg N/L)
Raw synthetic wastewater	–	–	–	–	150 ± 7	200 ± 8	20 ± 1	<2
I	1 ~ 90	100	11	12	74 ± 6.3	100 ± 9.5	9.2 ± 1.0	30 ~ 40
II	91 ~ 180	90	9	4	73 ± 4.1	102 ± 7.9	9.1 ± 1.2	30
III	180 ~ 310	90	6	2	72 ± 6.8	101 ± 5.7	10.3 ± 2.0	20 ~ 30

^a^The sludge retention time (SRT) was calculated by dividing the total reactor biomass by the average daily biomass wastage via effluent and sampling.

^b^It was measured immediately after feeding at the beginning of P release phase.

^c^The initial concentration of nitrate after nitrate dose at the beginning of P uptake phase was calculated theoretically by dividing the mass of nitrate dosage over the 10 L working volume of SBR.

**Table 2:** The overall performance of SBR at three experimental stages (average value  ±  standard deviation).

Parameters	Stage I	Stage II	Stage III
Volumetric acetate uptake rate (mg C/L/d)^a^	32 ± 6.5	76.5 ± 4.5	175 ± 15
Volumetric nitrate denitrification rate (mg N/L/d)^a^	17.5 ± 4.4	34.5 ± 4.8	65 ± 11
Volumetric sulfate reduction rate (mg S/L/d)^a^	14 ± 2.6	37.5 ± 6	85 ± 8.3
Volumetric P removal rate (mg P/L/d)^a^	2.3 ± 0.9	7.1 ± 2.6	20 ± 2.3
P content in the biomass (mg P/g VSS)	16 ~ 28	28 ~ 40	40 ~ 70
VSS concentration in the SBR (g VSS/L)	3.3 ± 0.2	3.5 ± 0.4	3.6 ± 0.3

^a^The volumetric rate was calculated based on {[[Measured initial concentration of the P release phase] − [Measured end concentration of decanting phase]] × [5 L influent/cycle] × [Cycle run numbers/day]/[10 L reactor working volume]} basis to describe the overall performance of the reactor.

**Table 3:** The comparison of DS-EBPR with conventional EBPR (average value  ±  standard deviation).

Parameters	This study in Stage III	Wu *et al*.^6^	Kuba *et al*.^7^
Operation temperature (°C)	22 ± 2	20 ± 1	22 ± 2
C/S mass ratio (mg C/mg S)	0.75	0.51 ± 0.04	NA^d^
P dosage (mg P/L)	10.3 ± 2.0	9.7 ± 1.5	7.5
Nitrate dosage (mg N/L)	26 ± 4.2	45	45
Volumetric acetate uptake rate (mg C/L/d)	175 ± 15^a^	58 ± 3.7	300
Volumetric sulfate reduction rate (mg S/L/d)	85 ± 8.3^a^	27 ± 3.6	NA^d^
Volumetric nitrate denitrification rate (mg N/L/d)	62 ± 11^a^	45	180
Volumetric P removal rate (mg P/L/d)	20 ± 2.3^a^	7.0 ± 1.4	30
Specific P removal rate (mg P/g VSS/d)	5.6 ± 0.6^b^	2.9 ± 0.7	10.7
P content in biomass (mg P/g VSS)	40 ~ 70	30 ~ 70	140 ~ 190
Cycle time (h)	9	24	6
P release time (h)	6	7.8	2.5
P uptake time (h)	2	13.8	3
SRT (day)	90	87 ± 11	20
Sludge yield (g VSS/g COD)	0.10 ± 0.02^c^	0.13 ~ 0.17	0.18 ~ 0.21

^a^The volumetric rate was calculated based on {[[Measured initial concentration of the P release phase] − [Measured end concentration of decanting phase]] × [5 L influent/cycle] × [Cycle run numbers/day]/[10 L reactor working volume]} basis to describe the overall performance of the reactor.

^b^The specific P removal rate was calculated based on {[[Measured initial concentration of the P release phase] − [Measured end concentration of decanting phase]] × [5 L influent/cycle] × [Cycle run numbers/day]/[10 L reactor working volume]/[MLVSS concentration}.

^c^The sludge yield was estimated based on the calculation of the sludge age by dividing the total reactor biomass by the average daily biomass wastage via effluent and sampling

^d^NA means not available.

**Table 4:** The energy balance analysis of DS-EBPR process.

Anaerobic P release			
	Reaction equation^14^,^15^	Inclusion (mmol/g VSS)	ATP (mmol/g VSS)
PHA generation	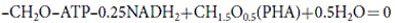	0.13	−0.13
Poly-P degradation	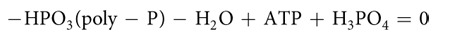	0.04	+0.04
Glycogen degradation	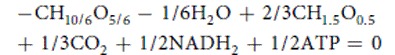	0.33	+0.17
Poly-S production^17^	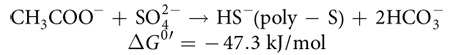	0.23	−0.09^a^
Anoxic P uptake			
PHA degradation	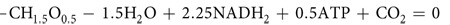	0.08	+0.04
Poly-P generation	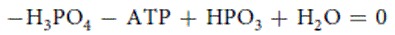	0.12	−0.12
Glycogen production	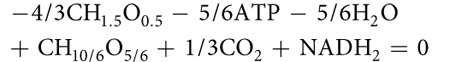	0.44	−0.36
Poly-S degradation^19^	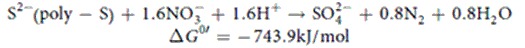	0.11	+0.66^a^

^a^ATP concentration was calculated based on the energy efficiency of 26% in anaerobic situation^18^, and the energy of 1 mol ATP was 31.4 kJ.
