# Supplementary material for: Effects of carbon-to-sulfur (C/S) ratio and nitrate (N) dosage on Denitrifying Sulfur cycle-associated Enhanced Biological Phosphorus Removal (DS-EBPR)
Source: Sci Rep. 2016 Mar 17;6:23221. doi: 10.1038/srep23221 (PMC4794707; doi:10.1038/srep23221)
Supplement: Supplementary Information [file srep23221-s1.pdf]

Supplementary Information For:

Effects of carbon-to-sulfur (C/S) ratio and nitrate (N) dosage on Denitrifying Sulfur cycle-associated Enhanced Biological Phosphorus Removal (DS-EBPR)

Mei Yu[a,b], Hui Lu[a,b,*], Di Wu[c], Qing Zhao[d], Fangang Meng[a,b], Yudan Wang[e], Xiaodi Hao[f] and Guang-Hao Chen[a,c,f]

[a] School of Environmental Science and Engineering, Sun Yat-sen University, Guangzhou, 510275, China

[b] Guangdong Provincial Key Laboratory of Environmental Pollution Control and Remediation Technology (Sun Yat-sen University), Guangzhou, 510275, China

[c] Department of Civil and Environmental Engineering, The Hong Kong University of Science and Technology, Clear Water Bay, Hong Kong, China

[d] School of Civil Engineering, Guangzhou University, Guangzhou, China

[e] Shenzhen Fengrun Environmental Technology Co., Ltd, Shenzhen, China

[f] Key Laboratory of Urban Stormwater System and Water Environment -MoU/R and D Centre for Sustainable Wastewater Treatment, Beijing University of Civil Engineering and Architecture, Beijing 100044, P. R. of China

[*]Corresponding author. Email address: lvhui3@mail.sysu.edu.cn

List of Supplementary Information

SI 1: PHA, poly-S and glycogen measurements

SI 2: DNA extraction, PCR amplification, pyrosequencing and date analysis

SI 3: Energy balance based on the metabolic model and the Gibbs free energy of chemical reaction

Table S1 The composition of synthetic wastewater

Table S2 The sequences and labels of the probes

Figure S1 Schematic diagrams: (a) SBR; and (b) Operation cycle of the SBR with feeding, anaerobic phosphorous release (P release), anoxic phosphorous uptake (P uptake), settling and decanting.

Figure S2 P release, P uptake and P removal performance of the SBR

Figure S3 The 454-pyrosequencing analysis at class level for the sludge sample taken from the SBR on Day 200.

Figure S4 Schematic draw of influent C/S ratio batch test

Figure S5 Schematic draw of nitrate dosage batch test

Supplementary Information 1:

PHA, poly-S and glycogen measurements

To determine the concentration of poly-β-hydroxyalkanoates (PHA), glycogen, and poly-S in the sludge of the SBR, about 10 mL mixed sludge samples were added to centrifuge tubes containing 2 drops of formaldehyde for biomass inactivation. The mixed sludge samples were then centrifuged, washed for three times with ultrapure water, and lyophilized 48 h in a Freeze Drier (Christ Freeze Dryers).

(1) PHA was determined with high performance liquid chromatography (HPLC) proposed by De Gelder et al.[1] and optimized. Approximately 15 mg pre-weighted pellets were added to 10 mL screwed-cap glass digestion tube, then added 0.5 mL 98% H_2SO_4 and 0.5 mL 100% methanol in turn. Six standards were composed of 0~2 mg of R-3-hydroxybutyric (3HB) and R-3-hydroxyvaleric acid (3HV) copolymer (12% HV, Sigma-Aldrich). The samples and standards were all digested for 2 h at 105 ^oC to form methyl crotonate, and then cooled down to room temperature. 9 mL of ultrapure water was added and mixed. The suspension was filtrated with a 0.22 μm Jin Teng nylon syringe filter. The methyl crotonate was determined by HPLC (Dionex Ultimate 3000) equipped with a C18 column (150mm×2.1μm×3μm) (Thermofisher).

(2) The poly-S was determined by using the sulfite method[2], which employed conversion of poly-sulfur and poly-sulfide to thiosulfate at high pH and 60 ^oC: $S^0 + SO_3^{2-} \rightarrow S_2O_3^{2-}$. About 15 mg pre-weighted dried solids were re-suspended in 8.35 mL of ultrapure water aerated with nitrogen. 1.5 mL of Na_2SO_3 solution (15% (w/v) solution in 1mM Na_2EDTA, made up with ultrapure water aerated with nitrogen) and 0.15 mL of 1 N NaOH was added to the suspension. Samples in tightly sealed 10 mL glass tube were incubated for 20~24 h in a 60 ^oC water bath. After the incubation, samples were cooled down to room temperature. The suspension was filtrated with a 0.22 μm Jin Teng PES syringe filter, and 1 mL the filtrate was transferred into an air-tight vial with 9 mL of ultrapure water, mixed for thiosulfate analysis using ion chromatograph (Dionex ICS-900). The final concentration of poly-S was determined from the thiosulfate concentration based on the aforesaid stoichiometric equation.

(3) Glycogen was analyzed by using the anthrone method according to Jenkins et al.[3]. About 5 mg of pre-weighted dried solids and 1 mL of 30% KOH (w/v) were added into a screwed-cap glass digestion tube which was digested at 100 ^oC for 3 h, and then cooled down to room temperature. 2 mL of distilled water and 6 mL of ice-cold ethanol (95%) were added to precipitate glycogen. The tube was then centrifuged at 4500 rpm for 15 min for decanting the supernatant. The pellets were dried at 60 ^oC in a drying oven (Binder), and the dried

pellets were re-suspended in 1 mL ultrapure water to solubilize the glycogen. The stock solution of glucose standard was prepared by adding 100 mg of glucose and 150 mg benzoic acid to 100 mL of ultrapure water, and then diluted at 1:10 with ultrapure water. The anthrone reagent solution was prepared by dissolving 200 mg of anthrone into 5 mL of absolute ethanol (100%), diluted to 100 mL with 75% sulfuric acid (v/v). 1mL of re-suspended pellets and the standards were added into 5 mL of chilled anthrone reagent solution, followed by heating in a boiling water bath for 10 min, then chilled in an ice-water bath. Absorbance of the final solution was measured at 625 nm through a UV-VIS spectrophotometer (Techcomp UV2300II).

Supplementary Information 2:

DNA extraction, PCR amplification, pyrosequencing and date analysis

The sludge sample was taken from SBR at day 200 of Stage III to analyse the microbial community. The sample was stored at -80 ^{o}C until the DNA extraction. The bacterial genomic DNA was extracted by using the PowerSoil DNA extraction kit (MO BIO Laboratories, Inc., Carlsbad, CA) following the manufacturer's instruction. The quality and quantity of the extracts were determined using a Nanodrop 1000 spectrophotometer (Thermo Fisher Scientific, Waltham, 190 MA) and then stored at -20 ^{o}C until use.

Fragments of the 16S rRNA gene were amplified by PCR using barcoded universal bacteria primers 8F and 533R targeting the V1-V3 hypervariable regions[4]. The primers were modified by adding an 8-nucleotide barcode (5'-GCGTCGTGTC-3') and corresponding pyrosequencing adaptors. The final sequences of the primers are 8F (5'-AGAGTTTGATCCTGGCTCAG-3') and 533R (5'-TTACCGCGGCTGCTGGCAC-3'). A 100 μL PCR reaction mixture contained 5 U of Pfu Turbo DNA polymerase (Stratagene, La Jolla, CA, USA), 1 x Pfu reaction buffer, 0.2 μM of dNTPs (TaKaRa, Dalin, China), 0.1 μM of each primer and 20 ng of genomic DNA template. PCR was performed with a thermal cycler (Bio-Rad, USA) under the following conditions: initial denaturation at 94 ^{o}C for 5 min; 30 cycles at 94 ^{o}C for 30 s, 53 ^{o}C for 30 s and 72 ^{o}C for 45 s; and a final extension at 72 ^{o}C for 10 min. The PCR products were purified using the TaKaRa Agarose Gel DNA Purification Kit (TaKaRa, China) and quantified with the NanoDrop device. The purified 16S amplicons were pyrosequenced using the ROCHE 454 FLX Titanium platform (Roche, Basel, Switzerland) at the National Human Genome Centre of China at Shang Hai, China (CHGC).

After filtering of the low quality sequences, FASTA files were generated from the resultant sequences according to the barcodes of individual samples. The sequences were then aligned using the software Mothur ver. 1.17.0[5] and the distance matrix was produced. Operational taxonomic unit (OUT) was determined at the levels of 90, 95 and 97% similarities. Rarefaction curves and the diversity indices (ACE and Chao 1) were determined based on the calculated OTUs using the same software. For the taxonomy-based analysis, the representative sequences from each OUT were subjected to the Ribosomal Database Project (RDP)[6], the National Centre for Biotechnology Information (NCBI) BLAST[7], and the Greengenes database[8].

Supplementary Information 3:

Energy balance based on the metabolic model and the Gibbs free energy of chemical reaction

Since the Gibbs free energy of PHA (i.e. acetyl-CoA, $\Delta G^{0'}_{acetyl-CoA} = -30.9\ kJ/eeq$)[9] and that of poly-S ($\Delta G^{0'}_{S^0/HS^-} = -26.1\ kJ/eeq$)[10] are quite close, the catabolic energy was considered identical between these two stored electron sources.

Based on the knowledge of metabolic model developed by Smolders et al.[11, 12], the energy changes of PHA and poly-S in P release and P uptake phases were estimated.

In P release phase:

Acetate uptake and storage as PHA

$$-CH_2O - ATP - 0.25NADH_2 + CH_{1.5}O_{0.5} + 0.5H_2O = 0$$

With PHA production 0.13 mmol/g VSS, ATP was consumed 0.13 mmol/g VSS.

Poly-P degradation for ATP production

$$-HPO_3 - H_2O + ATP + H_3PO_4 = 0$$

With 0.04 mmol/g VSS ploy-P degradation, ATP was yielded 0.04 mmol/g VSS.

Glycogen degradation

$$-CH_{10/6}O_{5/6} - 1/6\,H_2O + 2/3\,CH_{1.5}O_{0.5} + 1/3\,CO_2 + 1/2\,NADH_2 + 1/2\,ATP = 0$$

With glycogen consumed 0.33 mmol/g VSS, ATP was yielded 0.17 mmol/g VSS. As a result, ATP was stored 0.08 mmol/g VSS.

Sulfate reduction[13]

$$CH_3COO^- + SO_4^{2-} \rightarrow HS^- + 2HCO_3^- \quad \Delta G^{0'} = -47.3\ kJ/mol$$

With poly-S was stored 0.23 mmol/g VSS, there was about 0.09 mmol/g VSS ATP (31.4 kJ/mol) stored, based on the energy efficiency of 26% in anaerobic situation[14]. Therefore, the rich energy was stored in poly-S in the system.

In P uptake phase:

PHA catabolism

$$-CH_{1.5}O_{0.5} - 1.5H_2O + 2.25NADH_2 + 0.5ATP + CO_2 = 0$$

The PHA was degraded 0.08 mmol/g VSS, ATP was yielded 0.04 mmol/g VSS.

Poly-P synthesis

$$-H_3PO_4 - ATP + HPO_3 + H_2O = 0$$

Poly-P was produced 0.12 mmol/g VSS, ATP was consumed 0.12 mmol/g VSS.

Glycogen production

$$-4/3\,CH_{1.5}O_{0.5} - 5/6\,ATP - 5/6\,H_2O + CH_{10/6}O_{5/6} + 1/3\,CO_2 + NADH_2 = 0$$

With 0.44 mmol/g VSS glycogen production, ATP consumed 0.36 mmol/g VSS. As a result, there was about 0.44 mmol/g VSS ATP needed.

Denitrification[15]

$$S^{2-} + 1.6NO_3^- + 1.6H^+ \rightarrow SO_4^{2-} + 0.8N_2 + 0.8H_2O \quad \Delta G^{0\prime} = -743.9kJ/mol$$

$$S^{2-} + 0.4NO_3^- + 2.4H^+ \rightarrow S^0 + 0.2N_2 + 1.2H_2O \quad \Delta G^{0\prime} = -191.0kJ/mol$$

$$S^0 + 1.2NO_3^- + 0.4H_2O \rightarrow SO_4^{2-} + 0.6N_2 + 0.8H^+ \quad \Delta G^{0\prime} = -547.6kJ/mol$$

With denitrification consumed 0.11 mmol/g VSS poly-S, there was 0.66 mmol/g VSS ATP yielded, as 26% of the denitrification energy was stored as ATP. Poly-S can supply the energy deficient of this system.

Based on the preliminary energy balance, the following was found: 1) the energy was rich when only considering the PHA as an energy stored for P release and glycogen degradation in P-Release phase; 2) with the catabolic energy yield from PHA, the energy was deficient for synthesis of poly-P, glycogen and bacteria growth, maintenance. This energy based reasoning show that poly-S can act as an energy source in the DS-EBPR process.

Nutrient solution		Mineral solution	
Component	Concentration (g/L)	Component	Concentration (mg/L)
Sodium acetate	52.1	$FeCl_3 . 6H_2O$	2000
NH_4Cl	9.46	H_3BO_3	200
K_2HPO_4	6.70	$CuSO_4$	50
KH_2PO_4	3.50	KI	80
$MgCL_2$	3.90	$MnSO_4 . H_2O$	250
$CaCl_2$	5.20	$ZnSO_4 . 7H_2O$	150
EDTA	1.00	$CoCl_2 . 6H_2O$	200

Table S1. The composition of synthetic wastewater. The synthetic wastewater contained appropriate amounts of nitrogen and macro-minerals by adding 10 mL/L nutrient solution and 2 mL/L trace mineral solution; Sulfate prepared from 887.5 mg/L sodium sulfate.

Sequence (5'-3')	Probe name	5 label	3 label
GCTGCCTCCCGTAGGAGT	EUB338I[1]	FITC	FITC
GCAGCCACCCGTAGGTGT	EUB338II[1]	FITC	FITC
GCTGCCACCCGTAGGTGT	EUB338III[1]	FITC	FITC
CCGTCATCTACWCAGGGTATTAAC	PAO462 [2]	CY3	CY3
CCCTCTGCCAAACTCCAG	PAO651 [2]	CY3	CY3
GTTAGCTACGGCACTAAAAGG	PAO846 [2]	CY3	CY3
ACTTCTGCCAGATTCCAC	TBD1419 [3]	CY3	CY3
CTCGGTACGTTCCGACGC	TBD121 [3]	CY3	CY3
TCCCAGTCTTTGAGGTAC	TMD131 [3]	CY3	CY3
CGGCGTCGCTGCGTCAGG	SRB385[d] [4]	CY5	CY5
CGGCGTTGCTGCGTCAGG	SRB385Db [4]	CY5	CY5

1. EUBMIX (EUB338I-III) for the detection of all bacteria[16];

2. PAOMIX (PAO462, PAO651 and PAO846) for the detection of the *Betaproteobacteria Accumulibacter* spp.[17];

3. DMIX (TBD1419, TBD121 and TMD131) for the detection of denitrifying bacteria[18];

4. SRBMIX (SRB385[d] and SRB385Dd) for the detection of most of sulfate reducing bacteria (SRB)[19, 20].

Table S2. The sequences and labels of the probes.

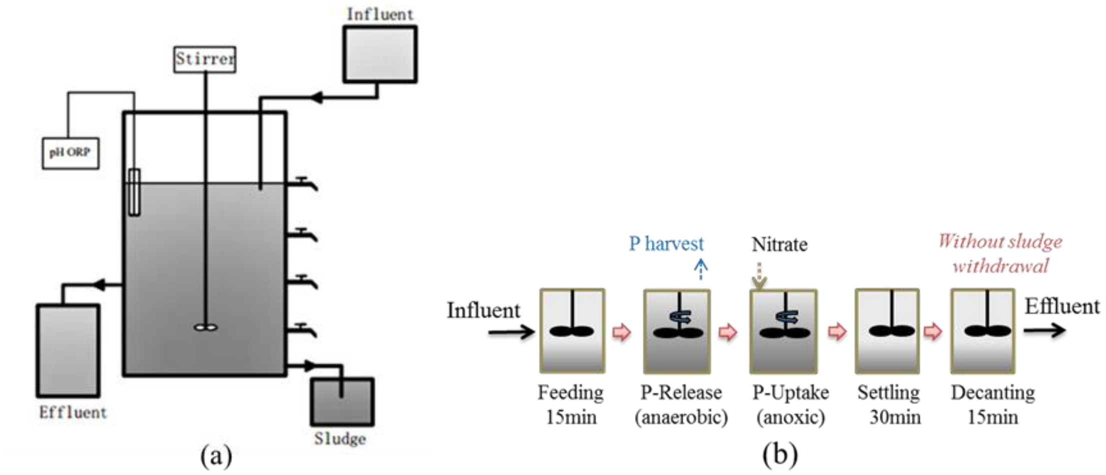

Figure S1. Schematic diagrams: (a) SBR; and (b) Operation cycle of the SBR with feeding, anaerobic phosphorous release (P release), anoxic phosphorous uptake (P uptake), settling and decanting.

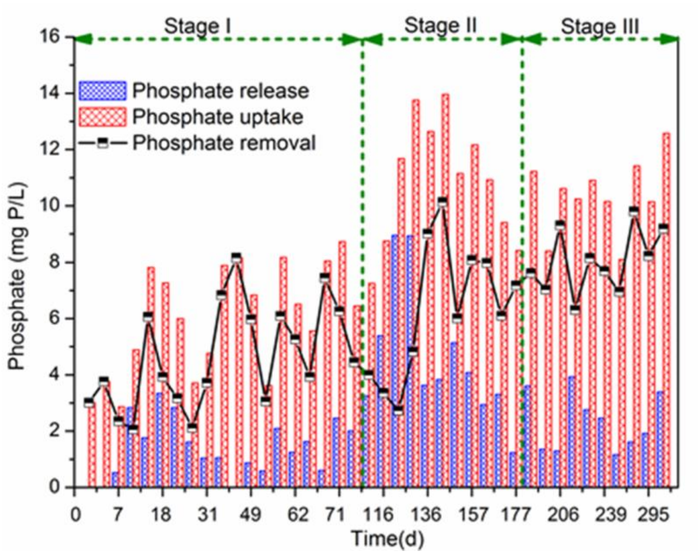

Figure S2. P release, P uptake and P removal performance of the SBR

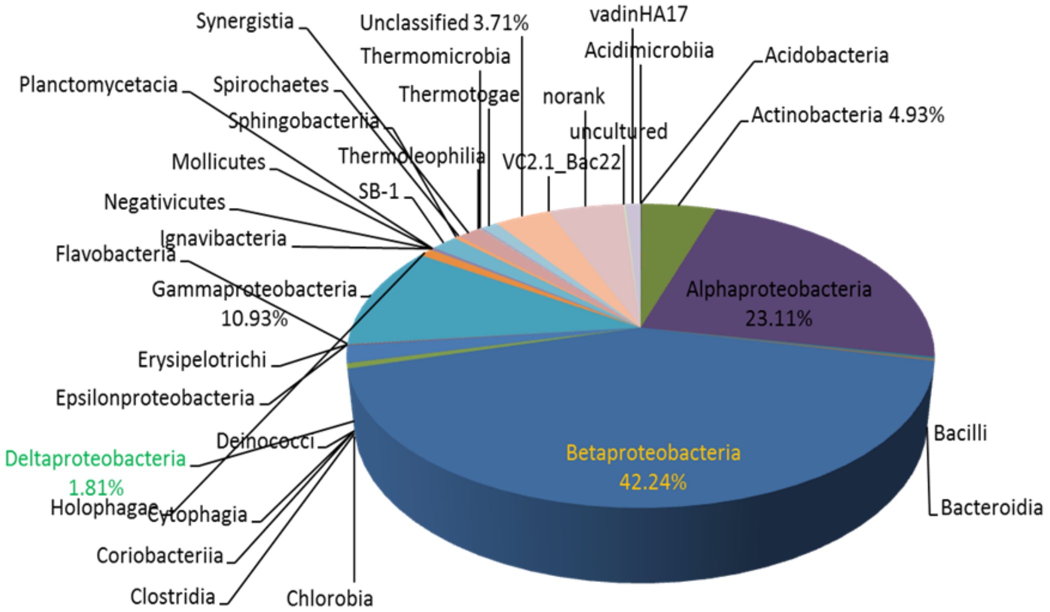

Figure S3. The 454-pyrosequencing analysis at class level for the sludge sample taken from the SBR on Day 200.

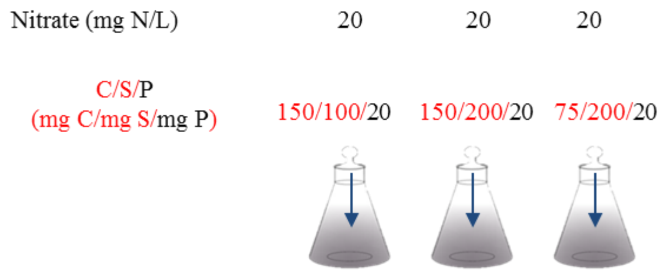

Figure S4. Schematic draw of influent C/S ratio batch test

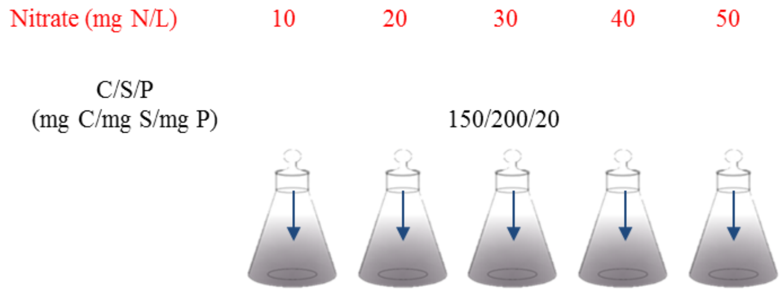

Figure S5. Schematic draw of nitrate dosage batch test

Supplementary Information Reference

1. De Gelder, J. et al. Monitoring poly (3-hydroxybutyrate) production in Cupriavidus necator DSM 428 (H16) with Raman spectroscopy. *Anal. Chem.* **80**(6), 2155-2160 (2008).

2. Jiang, G., Sharma, K. R., Guisasola, A., Keller, J. & Yuan, Z. Sulfur transformation in rising main sewers receiving nitrate dosage. *Water Res.* **43**(17), 4430-4440 (2009).

3. Jenkins, D., Richard, M. G., & Daigger, G. T. Glycogen fermentation in *Manual on the causes and control of activated sludge bulking and foaming. 2nd ed.* (eds Jenkins, D. et al.) 111-114 (Lewis, 1993).

4. Quince, C., Lanzen, A., Davenport, R. J. & Turnbaugh, P. J. Removing noise from pyrosequenced amplicons. *BMC Bioinformatics* **12**(1), 38 (2011).

5. Schloss, P. D. et al. Introducing mothur: open-source , platform-independent, community-supported software for describing and comparing microbial communities. *Appl. Environ. Microbiol.* **75**(23), 7537-7541 (2009).

6. Cole, J.R. et al. The Ribosomal Database Project: improved alignments and new tools for rRNA analysis. *Nucleic Acids Res.* **37** (Suppl.1), 141-145 (2009).

7. Johnson, M. et al. NCBI BLAST: a better web interface. *Nucleic Acids Res.* **36**(suppl 2), W5-W9 (2008).

8. DeSantis, T.Z. et al. Greengenes, a chimera-checked 16S rRNA gene database and workbench compatible with ARB. *Appl. Environ. Microbiol.* **72**(7), 5069-5072 (2006).

9. McCarty, P. L. Thermodynamic electron equivalents model for bacterial yield prediction: modifications and comparative evaluations. *Biotechnol. Bioeng.* **97**(2), 377-388 (2007).

10. Kelly, D. P. Biochemistry of the chemolithotrophic oxidation of inorganic sulphur. *Philosophical Transactions of the Royal Society of London. B: Biol. Sci.* **298**(1093), 499-528 (1982).

11. Smolders, G. J. F., Van der Meij, J., Van Loosdrecht, M. C. M. & Heijnen, J. J. Model of the anaerobic metabolism of the biological phosphorus removal process: stoichiometry and pH influence. *Biotechnol. Bioeng.* **43**(6), 461-470 (1994).

12. Smolders, G. J. F., Van der Meij, J., Van Loosdrecht, M. C. M. & Heijnen, J. J. Stoichiometric model of the aerobic metabolism of the biological phosphorus removal process. *Biotechnol. Bioeng.* **44**(7), 837-848 (1994).

13. Liamleam, W., Annachhatre, A. P. Electron donors for biological sulfate reduction. *Biotechnol. Adv.* **25**(5), 452-463 (2007).

14. Thauer, R. K., Jungermann, K. & Decker, K. Energy conservation in chemotrophic anaerobic bacteria. *Bacteriol. Rev.* **41**(1), 100 (1977).

15. Cardoso, R. B. et al. Sulfide oxidation under chemolithoautotrophic denitrifying conditions. *Biotechnol. Bioeng.* **95**(6), 1148-1157 (2006).

16. Daims, H., Brühl, A., Amann, R., Schleifer, K. H. & Wagner, M. The domain-specific probe EUB338 is insufficient for the detection of all Bacteria: development and evaluation of a more comprehensive probe set. *Syst. Appl. Microbiol.* **22**(3), 434-444 (1999).

17. Crocetti G. R. et al. Identification of polyphosphate-accumulating or-ganisms and design of 16S rRNA-directed probes for their detection and quantitation. *Appl. Environ. Microbiol.* **66**, 1175-1182 (2000).

18. Fernandez, N., Sierra-Alvarez, R., Field, J. A., Amils, R. & Sanz, J. L. Microbial community dynamics in a chemolithotrophic denitrification reactor inoculated with methanogenic granular sludge. *Chemosphere* **70**(3), 462-474 (2008).

19. Castro, H. F., Williams, N. H. & Ogram, A.. Phylogeny of sulfate-reducing bacteria. *FEMS Microbiol. Ecol.* **31**(1), 1-9 (2000).

20. Tonolla, M., Demarta, A., Peduzzi, S., Hahn, D. & Peduzzi, R. In situ analysis of sulfate-reducing bacteria related to Desulfocapsa thiozymogenes in the chemocline of meromictic Lake Cadagno (Switzerland). *Appl. Environ. Microbiol.* **66**(2), 820-824 (2000).
